# Childhood Obesity and Academic Performance: The Role of Working Memory

**DOI:** 10.3389/fpsyg.2017.00611

**Published:** 2017-04-19

**Authors:** Nan Wu, Yulu Chen, Jinhua Yang, Fei Li

**Affiliations:** ^1^Department of Psychology, Teachers’ College of Beijing Union UniversityBeijing, China; ^2^Department of Developmental and Behavioral Pediatrics, Shanghai Children’s Medical Center, Ministry of Education-Shanghai Key Laboratory of Children’s Environmental Health, School of Medicine, Shanghai Jiao Tong UniversityShanghai, China

**Keywords:** overweight and obese, children, academic performance, working memory, learning and memory

## Abstract

The present study examined the role of working memory in the association between childhood obesity and academic performance, and further determined whether memory deficits in obese children are domain-specific to certain tasks or domain-general. A total of 227 primary school students aged 10–13 years were analyzed for weight and height, of which 159 children (44 “obese,” 23 “overweight,” and 92 “normal weight”) filled out questionnaires on school performance and socioeconomic status. And then, all subjects finished three kinds of working memory tasks based on the digit memory task in 30 trials, which were image-generated with a series of numbers recall trial sets. After each trial set, subjects were given 5 s to recall and write down the numbers which hand appeared in the trial, in the inverse order in which they had appeared. The results showed there were significant academic performance differences among the three groups, with normal-weight children scoring higher than overweight and obese children after Bonferroni correction. A mediation model revealed a partial indirect effect of working memory in the relationship between obesity and academic performance. Although the performance of obese children in basic working memory tests was poorer than that of normal-weight children, they recalled more items than normal-weight children in working memory tasks involving with food/drink. Working memory deficits partially explain the poor academic performance of obese children. Those results indicated the obese children show domain-specific working memory deficits, whereas they recall more items than normal-weight children in working memory tasks associated with food/drink.

## Introduction

The prevalence of childhood obesity in China has tripled in the past 10 years, and more than 20% of 7–17 year-olds exceeded the 90th percentile of body mass index (BMI) in a national representative study in 2010 (Ma et al., 2012). Childhood obesity and overweight are associated with several metabolic and cardiovascular complications that have been well-documented (Daniels, 2006). Furthermore, childhood obesity is frequently associated with psychosocial issues (Krombholz, 2012); for example, overweight and obese children are more likely to have poor academic performance. Studies performed in the United States (Datar et al., 2004), Western Europe (Mikkila et al., 2003), South America (Campos et al., 1996), and Asia (Mo-suwan et al., 1999), showed significant and discouraging associations between childhood obesity and early academic outcomes. However, the mechanism underlying the association between obesity and poor academic performance remains unclear.

Although the exact association between obesity and poor academic performance remains to be defined, psychosocial factors, such as altered peer relationships and poor self-esteem (Wang and Veugelers, 2008; Zeller et al., 2008), have been suggested to influence the association between childhood obesity and school performance. These mental conditions may be among the factors mediating the poor performance of overweight or obese children in school. Recent research has also pointed to other cognitive abilities of the overweight and obese children (Tsal et al., 2016), including executive functioning (EF), that may affect their academic performance (Mond et al., 2007). EF skills are a set of cognitive processes that can shape multiple cognitive and behavioral outcomes across the life span, ranging from specific academic skills to overall school performance (Best et al., 2011). Working memory is one of the most central EF skills that emerge rapidly during the early childhood years and continues to develop throughout later childhood, and it is vital for preparing children to be successful in school (Liew, 2012). For example, individual differences in reading comprehension may reflect differences in working memory capacity, specifically in the trade-off between processing and storage functions (Best et al., 2011). Working memory is also an important predictor of math achievement. A study found that the working memory ability at 4 years of age could predict the math performance of 6 and 7 year-old children (Bull et al., 2008). Another study revealed that the particular difficulties of children with lower mathematical ability are related to their poor working memory, which results in problems controlling, regulating, switching and actively maintaining relevant mathematic information (Raghubar et al., 2010).

Given the vital role of working memory in children’s academic performance, it is worth focusing on the possible role of working memory in the association between childhood obesity and poor academic performance. Although there is no direct evidence showing the poor academic performance for obese children was related to the deficits in their working memory, many studies indicates that obese children have a poorer academic performance level than non-obese children. For example, Sabia (2007) studied a sample of American adolescents aged 14–17, she found that BMI was associated with lower grades. Another study also found an inverse relationship between BMI and academic achievement for school children aged 14–15 after controlling for parents’ educational level and parents’ occupation (Sigfúsdóttir et al., 2007). Shore et al. (2008) examined this issue with 271 6th graders and 301 7th graders and found overweight children showed lower scores on math and reading tests than non-overweight peers after adjusting for socioeconomic status. Furthermore, studies with adults and rats have found a link between obesity and deficits in learning and memory. For instance, compared with healthy weight adults, obese subjects show deficits in the one-back visual working memory task and executive function. Meanwhile, those obese adults consider themselves worse students (Stingl et al., 2012). A recent study found that a high calorie diet intake in obese rats resulted in both neurodegeneration and memory impairment (Trevino et al., 2015). Based on evidence from previous studies, obese children are more likely to develop a poor working memory, which might closely related to adverse academic outcomes. In addition, if the working memory is impaired in obese children, whether the deficit is domain-specific to certain tasks or domain-general needs to be further clarified.

The aim of the present study was to examine the role of working memory in the association between childhood obesity and academic performance, in which family socioeconomic status was fully considered, because (lower) socioeconomic status was more easily observed by other students and made an (stigma) effect on those obese children, and therefore contribute to poorer academic performance. It was hypothesized that overweight and obese children display a poorer working memory, which might lead to poorer academic performance than that of healthy weight children. In addition, different working memory tests were administered to explore whether the working memory deficit was domain-specific or domain-general for obese children.

## Materials and Methods

### Participants

A total of 227 primary school students ranging in age from 10 to 13 years were recruited by means of an advertisement asking for individuals to participate in a study relating to the relation between eating habits, dietary patterns and mental health [mean (*M*) = 12.07, standard deviation (*SD*) = 0.98]. All the participants reported themselves being in good health. Their weight and height were tested by the research assistant. According to the International Obesity Task Force guidelines, a leading manual on education and advocacy related to obesity, defines “overweight” for adolescents (ages 10–17) as a BMI greater than 25, whereas “obesity” is measured and defined as a BMI greater than 30. Of the 227 participants, 45 children were categorized as “obese (BMI ≥ 30),” and 23 children were categorized as “overweight (BMI ≥ 25).” Ninety-one children were categorized as “normal weight (18.5 ≤ BMI < 25),” and the remaining 69 children were categorized as “underweight (BMI < 18.5).” Therefore, the final participants in our study were 159 children, including 44 “obese” children, 23 “overweight” children, and 92 “normal weight” children (see **Table 1**).

**Table 1:** Descriptive statistics of the participants in the present study.

Cohort	*n*	Gender (*n*)		Age	
			
		Boys	Girls	Mean	*SD*
Obese group	44	30	14	12.38	1.22
Overweight group	23	17	6	11.78	1.00
Normal-weight group	92	40	52	11.93	0.92


The present experiment was approved by the Ethics Committee of Teachers’ College of Beijing Union University, and written consent was obtained from each student’s parents and guardian prior to the experiment.

### Measures

#### Working Memory Tasks

##### The digit span memory task

The digit span memory task used in the present study is the Chinese version of digit span subtest which taken from WAIS-IV (Zhang, 2009). The WAIS- IV consists of 10 primary subtests (vocabulary, information, similarities, digit span, arithmetic, block design, matrix reasoning, visual puzzles, coding, and symbol search) that yield four Index scores (verbal comprehension, perceptual reasoning, working memory, and processing speed) and an overall Full-Scale IQ (Wechsler, 2003). The digit span memory task is a classical measurement of immediate memory and working memory maintenance and manipulation.

The working memory materials used in the present study were image-generated (sized 15.2 cm × 6.3 cm) and presented on the center of screen with a series of numbers recall trial sets. Each trial set was presented at the rate of one number per second. After each trial set, subjects were given 5 s to recall and write down the numbers which hand appeared in the trial, in the inverse order in which they had appeared. All subjects were presented with three kinds of working memory materials based on the digit memory task. As the order of the tasks was counter-balanced between subjects, one third started the first test while one third started the second test and one third started the third working memory test.

**FIGURE 1:**
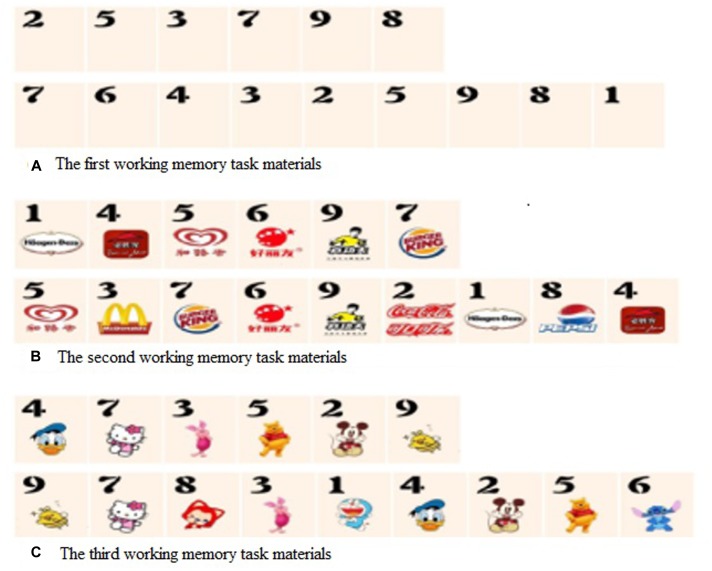
**Examples of the three kinds of working memory materials used in the study.** **(A)** Example of the typical digit memory task. **(B)** Example of the digit presented together with a food or drink mark. **(C)** Example of the digit presented together with a cartoon image. In each task, the participants were asked to recall and write down the first line and second line number in the inverse order.

The first test was the typical digit memory task (**Figure 1A**). Each subject was asked to recall and write down a number of digits in the inverse order that were previously displayed on the computer screen. The list began with two digits and increased sequentially until recall errors were made on at least one of the two trials. The increasing set of numbers’ backward recall can assess working memory. The participants’ performance was calculated based on the number of digits that they were able to recall without mistakes. In the second working memory task (**Figure 1B**), each digit was presented together with a food or drink mark, and only the number of digits that they were able to write down in reverse order without errors were calculated as working memory scores. In the third working memory task (**Figure 1C**), each digit was presented together with a cartoon image, and working memory scores were calculated from the numbers of digits that the subject could recall and write down correctly in reverse order. **Figure 1** shows the examples (6 and 9 number length) of three different working memory materials.

#### School Performance and Socioeconomic Status

For each participant, the Chinese, English, and mathematics scores of the previous year were collected and recorded as their academic achievement. Before the working memory test, children in the three groups were asked their family socioeconomic status including: (1) household income; (2) parents’ school diploma and higher education; (3) parents’ occupation. And the socioeconomic status data was checked again by students’ enrollment to school at the beginning of the academic year.

### Procedure

Upon arrival, the procedures were explained and written informed consent was obtained. The participants were seated in a quiet room and the demographic questionnaire was administered by a research assistant, and the data on weight, height as well as the academic performance were collected. All participant were then randomly divided into three group to match the orders of the three working memory tasks. At the beginning of the test, each participant was taken into a separate room and completed the working memory tasks on the computer screen conducted by a female experimenter. A small gift as payment was handed to each participant at the end of the experiments.

## Results

GLM univariance analysis (with SES effects parsed out) was applied to test school performance differences among the three groups, and a multilevel modeling (MLM) paradigm (Preacher et al., 2010) was administered to explore the role of working memory in the association between childhood obesity and academic performance. An analysis of variance (ANOVA) and planned *post hoc* (Bonferroni) tests were used to investigate group effects on the three working memory tasks.

### School Performance Differences among the Three Groups and Relationship with Working Memory

**FIGURE 2:**
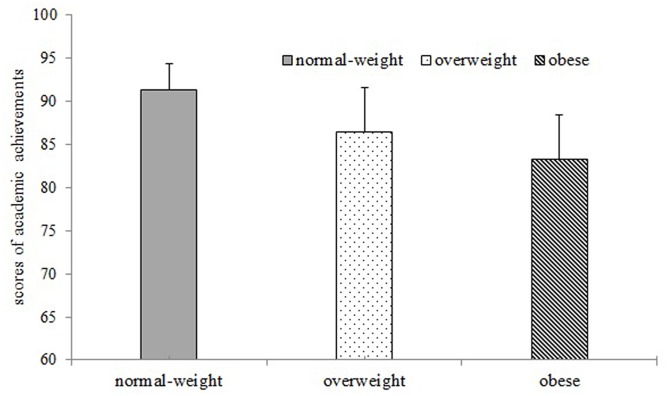
**Normal-weight children had better academic performance than obese children**.

The association between obesity and academic performance was tested using GLM univariance analysis with SES factors (income and parental education status) as covariates. Because of the large differences in sample sizes across the three groups (44 obese children, 23 overweight children, and 92 normal-weight children), Levene’s Test was conducted to check whether the variances of the three groups being compared are equal. The results showed Levene’s Test was consistent with the homogeneity of variances assumption, *F*(2,156) = 1.87, *p* = 0.158. Further GLM univariance analysis showed significant academic performance differences among normal-weight, overweight, and obese children (**Figure 2**), *F*(2,153) = 16.73, *p* < 0.001, η^2^ = 0.18. Normal-weight children scored higher than overweight (*p* = 0.02) and obese children (*p* < 0.001) after Bonferroni correction. There was no significant difference between overweight children and obese children (*p* = 0.34).

**FIGURE 3:**
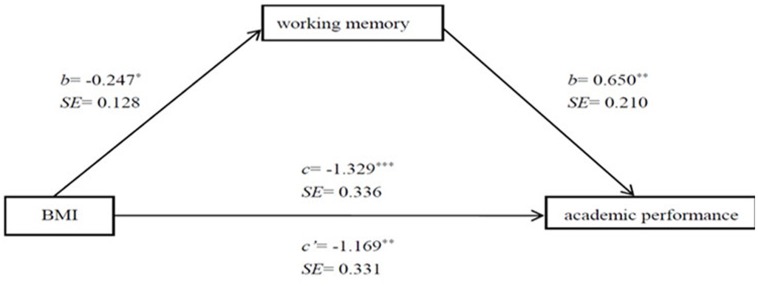
**Mediation model of BMI, working memory and academic performance.** For the path from BMI to academic performance, the upper *c*-weight indicated the effect without working memory in the model and the lower *c′*-weight indicated the effect with working memory in the model. *^∗^p* < 0.05, *^∗∗^p* < 0.01, *^∗∗∗^p* < 0.001.

To explore the possible role of working memory in the association between childhood obesity and academic performance, we used a mediation model, in which working memory was hypothesized as mediator. The results of this model suggested that BMI was negatively associated with academic performance (*b* = -1.329, *SE* = 0.336, *p* < 0.001). A full path model was then estimated to test the mediation hypothesis. The results of the model are shown in **Figure 3**. A higher BMI predicted a poorer working memory (*b* = -0.247, *SE* = 0.128, *p* = 0.045), and a poorer working memory was associated with a lower level of academic performance (*b* = 0.650, *SE* = 0.210, *p* = 0.002). Furthermore, the link between BMI and academic performance remained significant after working memory was added to the model, and the strength of this association decreased by 7% (*b* = -1.196, *SE* = 0.331, *p* < 0.001). The significance of the indirect effect was tested using a model-based bootstrap with 5000 samples from the original data, which revealed a partial indirect effect of working memory, with a 95% confidence interval of -0.328, -0.024 (Preacher and Hayes, 2008). The absence of zero in the confidence interval for the total indirect effect of BMI on academic performance as mediated by working memory was significant (Sobel’s *Z* = -1.561, *p* = 0.034). The results are summarized in **Figure 3**.

### Comparison of Working Memory Performance in the Three Tasks

**FIGURE 4:**
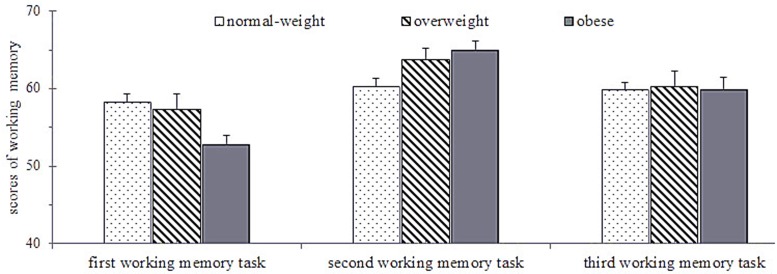
**Comparison of three groups’ working memory performance during each testing stage**.

Analysis of variance was followed by the Bonferroni significant difference test to explore whether the poor working memory ability in obese children was domain-general or domain-specific. Similarly, we conducted the Levene’s Test to check whether the variances of the three groups being compared are equal. The results showed Levene’s Test was consistent with the homogeneity of variances assumption on the first working memory task (*F* = 1.315, *p* = 0.73) and the second working memory task (*F* = 1.062, *p* = 0.348) as well as the third working memory task (*F* = 0.728, *p* = 0.484). Further ANOVA analysis showed there was a main group effect on the first and second working memory tasks [*F*(2,156) = 4.452, *p* = 0.012, η^2^ = 0.06; *F*(2,156) = 4.343, *p* = 0.015, η^2^ = 0.05]. The Bonferroni test showed that the normal weight group (*M* = 57.14, *SD* = 10.29) scored higher than the obese group (*M* = 52.70, *SD* = 8.78) in the basic working memory test (*p* = 0.043). By contrast, the obese group (*M* = 64.86, *SD* = 8.28) performed better than the normal weight group (*M* = 60.34, *SD* = 8.78) in the second working memory task with food/drink marks (*p* = 0.006). There were no significant differences among the three groups in the third working memory task [*F*(2,156) = 0.12, *p* = 0.89]. The mean recall scores in the three working memory tasks are shown in **Figure 4**.

## Discussion

There were two important findings in the present study: first, the results showed that childhood obesity was associated with poor academic performance, and that this poor school performance among obese children was partially due to their poor basic working memory ability. The second finding is that although obese children performed worse than normal-weight children in basic working memory tests, they recalled more items than normal-weight children in the working memory task with food/drinks marks. This demonstrated that obese children were more sensitive to the memory materials with food/drink marks, indicating that the working memory deficit for obese children might be domain-specific.

The association between childhood obesity and poor school performance, which is discouraging, has been demonstrated in various cultures. A review of nine studies performed in the United States, Western Europe, South America, and Asia suggested a consistent and significant association (Taras and Potts-Datema, 2005). Another recent longitudinal study indicated that weight status during the fifth grade predicted lower levels of academic achievement in eighth grade, and this relation was maintained after controlling for numerous potential confounding variables, including socioeconomic indicators, physical activity, and TV watching (Purtell and Gershoff, 2015). Among those confounding variables, socioeconomic characteristics is more easily observable by other students, and its potential association with worse academic performance can contribute to the stigma of overweight, which might inverse leads to poor academic performance at school. While in our study, after controlling for socioeconomic status, we still found significant relationship between obesity and academic performance. The present study was based on Chinese children and provided consistent findings of poor school performance among children who are obese.

A possible mechanism for the association between childhood obesity and poor academic performance was identified in our study, as our results showed that working memory partially mediated the link between childhood obesity and poor academic performance. Working memory represents the basic cognitive system in that it underlies the capacity to remember moment to moment information on activities and serves to allocate cognitive processing capacity to the various other cognitive systems, according to need and importance. There has been a clear generalization concerning the important positive relationship between math, literature learning, and the demand on working memory. For example, working memory is increasingly involved in arithmetic learning and math problem solving (Swanson, 2014). In the process of Chinese and English learning, working memory for word recall and reading comprehension is also in high demand (Bull et al., 2008; Best et al., 2011). Furthermore, previous studies demonstrated that compared with healthy weight individuals, obese children show significant impairments in working memory (Francis and Stevenson, 2011; Stingl et al., 2012; Fitzpatrick et al., 2013; Coppin et al., 2014), which is closely related to their poor academic performance (Judge and Jahns, 2007). The current data confirm previous work in that working memory partially explained the obese children’s disadvantage in academic learning.

Although the poor academic performance of obese children was partially due to their impaired working memory ability, whether the working memory deficit for obese children was domain-specific or domain-general remains unclear. Our study design based on three different working tasks revealed that obese children recall more items than normal-weight children in the working memory task with food/drink marks, whereas the obese group performed worse than the normal-weight group in the basic working memory task, indicating that the deficit in learning and memory for obese children was domain-specific. This also suggests that the learning and memory processes in obese children were different from those of normal weight children, as obese children were more sensitive to the memory tasks with food/drink marks. Prior studies of explicit learning and memory in adults showed that obese individuals show a stronger responsiveness to external cues related to food; for example, obese women completed significantly more food-and shape-related words than other groups in the explicit memory tasks (Docteur et al., 2008). The proposed mechanism was that the presence of food cues improve the memory efficiency of obese individuals through imagery, in which external information is quickly and vividly organized into an internal representation and stored as a spatial schema (Fioravanti et al., 2004). Consistent with prior studies, we observed that obese children recalled items in a different way, as their processing of information with food/drink marks was more efficient than that of normal-weight children.

The present study had several limitations. Poor academic performance in obese children is influenced by multiple factors; therefore, working memory plays a limited role in explaining the disadvantages of obese children regarding academic performance. It is also important to note that the sample sizes of the current study were not optimal. Thus, future studies with larger sample sizes and consideration of additional factors will help clarify the association between childhood obesity and poor school performance. Despite these limitations, to the best of our knowledge, this is the first study demonstrating the role of working memory in the association between childhood obesity and academic performance in the Chinese population.

## Conclusion

The present study found the association between childhood obesity and poor academic performance in Chinese children and indicated that this disadvantage in school performance among obese children was partially due to their poor basic working memory ability. Furthermore, the current findings extend the body of evidence related to learning and memory processes in obese children by indicating that the deficit in working memory for obese children might be domain-specific.

## Author Contributions

NW designed the experiment, collected data and prepared the manuscript. YC collected the data and made data analysis. JY corrected the whole language of the manuscript and made final approval. FL gave technique supports and valuable suggestions in experiment designing.

## Conflict of Interest Statement

The authors declare that the research was conducted in the absence of any commercial or financial relationships that could be construed as a potential conflict of interest.

## References

[B1] Best J. R. Miller P. H. Naglieri J. A. (2011). Relations between executive function and academic achievement from ages 5 to 17 in a large, representative national sample. *Learn. Individ. Differ.* 21 327–336. 10.1016/j.lindif.2011.01.007 21845021 PMC3155246

[B2] Bull R. Espy K. A. Wiebe S. A. (2008). Short-term memory, working memory, and executive functioning in preschoolers: longitudinal predictors of mathematical achievement at age 7 years. *Dev. Neuropsychol.* 33 205–228. 10.1080/87565640801982312 18473197 PMC2729141

[B3] Campos A. L. Sigulem D. M. Moraes D. E. Escrivao A. M. Fisberg M. (1996). Intelligent quotient of obese children and adolescents by the Weschler scale. *Rev. Saude Publica* 30 85–90. 9008926 10.1590/s0034-89101996000100011

[B4] Coppin G. Nolan-Poupart S. Jones-Gotman M. Small D. M. (2014). Working memory and reward association learning impairments in obesity. *Neuropsychologia* 65 146–155. 10.1016/j.neuropsychologia.2014.10.004 25447070 PMC4259845

[B5] Daniels S. R. (2006). The consequences of childhood overweight and obesity. *Future Child.* 16 47–67. 10.1353/foc.2006.0004 16532658

[B6] Datar A. Sturm R. Magnabosco J. L. (2004). Childhood overweight and academic performance: national study of kindergartners and first-graders. *Obes. Res.* 12 58–68. 10.1038/oby.2004.9 14742843

[B7] Docteur A. Defrance C. Raison J. Urdapilleta I. (2008). Implicit and explicit memory bias for food, shape and body parts related words in obese and normal weight females. *Psychol. Lett. Behav. Brain Cogn.* 5 52–62. 10.1037/0021-843x.107.2.193

[B8] Fioravanti M. Polzonetti C. M. Nocca D. Spera G. Falcone S. Lazzari R. (2004). Emotional activation of obese and normal women due to imagery and food content of verbal stimuli in a memory task. *Eat. Behav.* 5 47–54. 10.1016/s1471-0153(03)00063-1 15000953

[B9] Fitzpatrick S. Gilbert S. Serpell L. (2013). Systematic review: are overweight and obese individuals impaired on behavioural tasks of executive functioning? *Neuropsychol. Rev.* 23 138–156. 10.1007/s11065-013-9224-7 23381140

[B10] Francis H. M. Stevenson R. J. (2011). Higher reported saturated fat and refined sugar intake is associated with reduced hippocampal-dependent memory and sensitivity to interceptive signals. *Behav. Neurosci.* 125 943–955. 10.1037/a0025998 22023100

[B11] Judge S. Jahns L. (2007). Association of overweight with academic performance and social and behavioral problems: an update from the early childhood longitudinal study. *J. Sch. Health* 77 672–678. 10.1016/1054-139X(94)90514-2 18076412

[B12] Krombholz H. (2012). The motor and cognitive development of overweight preschool children. *Early Years* 32 61–70. 10.1080/09575146.2011.599795

[B13] Liew J. (2012). Effortful control, executive functions, and education: bringing self-regulatory and social-emotional competencies to the table. *Child. Dev. Perspect.* 6 105–111. 10.1111/j.1750-8606.2011.00196.x

[B14] Ma J. Cai C. H. Wang H. J. (2012). Prevalence and trends of overweight and obesity in Chinese children from 1985 to 2010. *J. Preven. Med.* 46 776–780. 10.3760/cma.j.issn.0253-9624.2015.10.001 23157879

[B15] Mikkila V. Lahti-Koski M. Pietinen P. Virtanen S. M. Rimpela M. (2003). Associates of obesity and weight dissatisfaction among Finnish adolescents. *Public Health Nutr.* 6 49–56. 10.1079/phn2002352 12581465

[B16] Mond J. M. Stich H. Hay P. J. Kraemer A. Baune B. T. (2007). Associations between obesity and developmental functioning in pre-school children: a population-based study. *Int. J. Obes.* 31 1068–1073. 10.1038/sj.ijo.0803644 17471298

[B17] Mo-suwan L. Lebel L. Puetpaiboon A. (1999). School performance and weight status of children and young adolescents in a transitional society in Thailand. *Int. J. Obes. Relat. Metab. Disord.* 23 272–277. 10.1038/sj.ijo.0800808 10193872

[B18] Preacher K. J. Hayes A. (2008). Asymptotic and resampling strategies for assessing and comparing indirect effects in multiple mediator models. *Behav. Res. Meth.* 40 879–891. 10.3758/brm.40.3.879 18697684

[B19] Preacher K. J. Zyphur M. J. Zhang Z. (2010). A general multilevel SEM framework for assessing multilevel mediation. *Psychol. Methods* 15 209–233. 10.1037/a0020141 20822249

[B20] Purtell K. M. Gershoff E. T. (2015). Fast food consumption and academic growth in late childhood. *Clin. Pediatr.* 54 871–877. 10.1177/0009922814561742 PMC8887837 25480321

[B21] Raghubar K. P. Barnes M. A. Hecht S. A. (2010). Working memory and mathematics: a review of developmental, individual difference, and cognitive approaches. *Learn. Individ. Differ.* 20 110–122. 10.1016/j.lindif.2009.10.005

[B22] Sabia J. J. (2007). The effect of body weight on adolescent academic performance. *South. Econ. J.* 73 871–900. 10.2307/20111933

[B23] Shore S. M. Sachs M. L. Lindicker J. R. Brett S. N. Wright A. R. Libonati J. R. (2008). Decreased scholastic achievement in overweight middle school students. *Obesity* 16 1535–1538. 10.1038/oby.2008.254 18451772

[B24] Sigfúsdóttir I. D. Kristijánsson A. L. Allegrante J. P. (2007). Health behavior and academic achievement in Icelandic school children. *Health Edu. Res.* 22 70–80. 10.1093/her/cyl044 16766605

[B25] Stingl K. T. Kullmann S. Ketterer C. Heni M. Haring H.-U. Fritsche A. (2012). Neuronal correlates of reduced memory performance in overweight subjects. *Neuroimage* 60 362–369. 10.1016/j.neuroimage.2011.12.012 22197786

[B26] Swanson H. L. (2014). Working memory and phonological processing as predictors of children’s mathematical problem solving at different ages. *Mem. Cogn.* 32 648–661. 10.3758/BF03195856 15478759

[B27] Taras H. Potts-Datema W. (2005). Obesity and student performance at school. *J. Sch. Health* 75 291–295. 10.1111/j.1746-1561.2005.tb07346.x 16179079

[B28] Trevino S. Aguilar-Alonso P. Hernandez J. A. F. Brambila E. Guevara J. Flores G. (2015). A high calorie diet causes memory loss, metabolic syndrome and oxidative stress into hippocampus and temporal cortex of rats. *Synapse* 69 421–433. 10.1002/syn.21832 26073877

[B29] Tsal C. L. Chen F. C. Pan C. Y. Tseng Y. T. (2016). The neurocognitive performance of visuospatial attention in children with obesity. *Front. Psychol.* 7 1033 10.3389/fpsyg.2016.01033 PMC4933706 27458421

[B30] Wang F. Veugelers P. J. (2008). Self-esteem and cognitive development in the era of the childhood obesity epidemic. *Obes. Rev.* 9 615–623. 10.1111/j.1467-789x.2008.00507.x 18647242

[B31] Wechsler H. C. (2003). *Wechsler Intelligence Scale for Children*, 4th Edn San Antonio, TX: Pearson.

[B32] Zeller M. H. Reiter-Purtill J. Ramey C. (2008). Negative peer perceptions of obese children in the classroom environment. *Obesity* 16 755–762. 10.1038/oby.2008.143 18379560 PMC2713023

[B33] Zhang H. C. (2009). The reversion of WISC- IV Chinese version. *Psychol. Sci.* 32 1177–1179.

